# Best timing of bilateral total hip arthroplasty – an analysis of revision and mortality rates from the German Arthroplasty Registry (EPRD)

**DOI:** 10.1186/s12891-024-07693-7

**Published:** 2024-08-02

**Authors:** Anne Postler, Paula Krull, Klaus-Peter Günther, Jörg Lützner, Oliver Melsheimer, Arnd Steinbrück, Jens Goronzy

**Affiliations:** 1 University Center of Orthopaedic, Trauma and Plastic Surgery, University Hospital Carl Gustav Carus, TUD Dresden University of Technology, Fetscherstr. 74, 01307 Dresden, Germany; 2 German Arthroplasty Registry (EPRD), Berlin, Germany

**Keywords:** Total hip arthroplasty, Bilateral, Same day, Simultaneous, Mortality, Revision

## Abstract

**Background:**

The burden of osteoarthritis (OA) in multiple joints is high and for patients with bilateral OA of the hip there is no clear recommendation about the indication for simultaneous (one-stage) bilateral total hip arthroplasty (THA) versus two-staged procedures. The purpose of this study was therefore to compare revision and mortality rates after different strategies of surgical timing in bilateral hip OA from the German Arthroplasty Registry (EPRD).

**Methods:**

Since 2012 22,500 patients with bilateral THA (including 767 patients with one-staged bilateral surgery and 11,796 patients with another separate procedures within one year after first THA) are documented in the registry. The patients who underwent simultaneous bilateral THA were matched with a cohort of 767 patients who underwent the second THA between 1 and 90 days postoperatively (short interval) and another cohort of 4,602 patients with THA between 91 and 365 days postoperatively (intermediate interval). Revision for all reasons and mortality rates were recorded. Cox regression was performed to evaluate the influence of different patient characteristics.

**Results:**

The cumulative 5-year revision rate for patients with simultaneous bilateral THA was 1.8% (95% CI 0.9–2.6), for patients with two-staged THA 2.3% (95% CI 1.0-3.6) in the short interval and 2.5% (95% CI 2.1–2.9) in the intermediate interval, respectively. In all three groups, patients who underwent THA in a high-volume center (≥ 500 THA per year) had a significant lower risk for revision (HR 0.687; 95% CI 0.501–0.942) compared to surgeries in a low-volume center (< 250 THA per year). There was no significant difference regarding cumulative mortality rates in the three cohorts. Higher age (HR 1.060; 95% CI 1.042–1.078) and severe comorbidities as reflected in the Elixhauser Score (HR 1.046; 95% CI 1.014–1.079) were associated with higher mortality rates after simultaneous THA.

**Conclusion:**

Simultaneous bilateral THA seems to be a safe procedure for younger patients with limited comorbidities who have bilateral end-stage hip OA, especially if performed in high-volume centers.

**Level of evidence:**

III.

## Introduction

Total hip arthroplasty (THA) is one of the most frequent surgical procedures and a very effective treatment option for advanced osteoarthritis of the hip, which decreases pain and improves function [1]. Evans et al. reported about an expected survival time of THA up to 25 years in around 58% of patients [2].

The burden of OA in multiple joints is high [3] and multiple surgeries and anesthesia procedures need a prolonged rehabilitation and recovery. Usually, those patients undergo staged THA and second surgery on the contralateral side adds a significant additional period of convalescence to the recovery time after initial THA. The alternative of one-staged, simultaneous bilateral THA on the same day is rather reserved for younger patients with less comorbidities [4], but risks and benefit are discussed controversially.

In 1971 Jaffe and Charnley reported about fifty cases of bilateral THA and the option to treat patients with bilateral one-staged simultaneous surgery [5]. Since that time the interest in simultaneous THA on the same day and during one single anesthetic procedure, is increasing. Some studies compared simultaneous bilateral THA versus unilateral THA [6–8] or versus staged bilateral surgery under two separate anesthetic procedures [9–14]. Most of them showed overall reduced hospital stay [12], faster rehabilitation and improved cost-effectiveness for simultaneous bilateral THA [13].

However, a national data base survey revealed greater risk for complication and higher rates of mortality after simultaneous bilateral THA [15]. In a recently published systematic review and meta-analysis a tendency towards fewer complications and lower total cost after simultaneous bilateral THA has been reported, but the authors highlight a necessity of further analyses, as the evidence from available studies is not sufficient [16].

In addition, most studies compared simultaneous THA, which is defined as THA on the same day during the same anesthetic procedure, with staged procedures in general. There is considerable heterogeneity, however, regarding the time interval between initial THA on one side and consecutive surgery on the contralateral side, which also might influence the outcome.

Aim of our study was therefore (1) to investigate the frequency and timing of bilateral THA in Germany and (2) to determine revision and mortality rates associated with simultaneous versus two-stage THA in two different, but exactly defined time intervals in appropriately matched cohorts from the German Arthroplasty Registry (EPRD).

## Methods

The EPRD started data acquisition in November 2012 and includes currently a total number of more than 2 million hip and knee replacements in its database. It covers primary and revision arthroplasty surgeries. Although participation is voluntarily, it covers about 70% of all hip and knee arthroplasties in Germany [17]. Once entered into the registry, the follow-up of an arthroplasty is nearly complete because data on revisions is obtained not only by hospitals, but additionally by health insurance companies. Demographic data such as age, sex and body mass index as well as comorbidities are documented. Death and revision data are obtained from health insurance companies on a regular basis [18].

From a total number of 308,473 patients with THA performed for hip OA (ICD-10 code diagnoses of M16.-), 22,500 could be identified as having a bilateral primary THA. In 11,796 cases both surgeries were performed within one year. Patients with a THA due to other diagnosis than OA (M16) or hemiarthroplasty were not included. Patients under the age of 18 years were also excluded.

Three different time intervals between both THA were chosen: (1) both procedures on exactly the same day (simultaneous), (2) staged surgery with an interval of one to 90 days between index surgery and second THA (short interval) and (3) staged surgery with an interval of 91 to 365 days between both procedures (intermediate interval). We identified 90 days as the staged interval as this is common the time frame for risk to return to normal. as the episode after essential recovery.

**Fig. 1:**
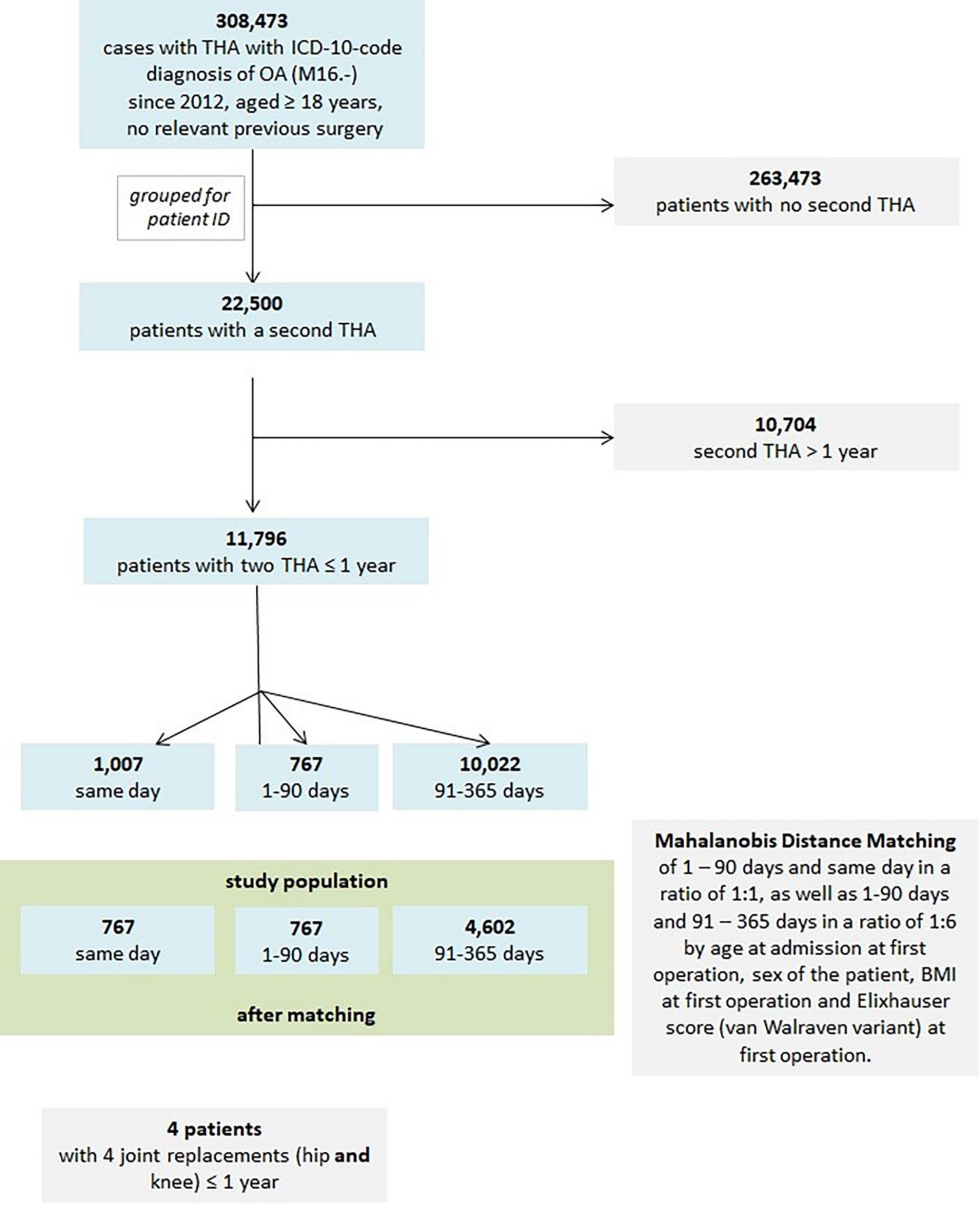
Flowchart of all patients and selection process. ICD-10-GM: International classification of diseases, 10th revision, German modification, OA: osteoarthritis

The three groups were suitable for a matched pair analysis, according to the time interval between both THA, simultaneous (*n* = 1,007; 8.5%), short interval (*n* = 767; 6.5%) and intermediate interval (*n* = 10,022; 85.0%), see flowchart, Fig. 1.

### Statistical analysis

There were significant differences regarding age, sex, BMI and comorbidities (weighted Elixhauser score) [19] between the three groups. To reduce the bias by these factors a Mahalanobis-Distance-Matching was used for short interval and simultaneous in a ratio of 1:1, as well as short and intermediate interval in a ratio of 1:6 by age at admission at first operation, patients’ sex, BMI at first operation and Elixhauser score (van Walraven variant) at first THA.

A perfect balance after matching could not be achieved for the variables age and BMI, because group sizes of simultaneous and intermediate interval only differed by 240 cases. However, these differences were small and can be considered clinically not relevant.

Data description was based on means and standard deviation (SD) for continuous variables and absolute and relative frequencies for categorical variables.

Cumulative incidences for the endpoints death of the patient and revision of arthroplasty were calculated with the Kaplan-Meier estimator for the matched dataset. A pairwise Log-Rank test with Holm´s correction for multiple testing was applied to identify intergroup differences. To evaluate the influence of different patient characteristics on revision and mortality a multivariate Cox regression was applied to the matched dataset. The model was started as a full model, including all variables that were available from the registry and discussed as relevant confounders. Included variables were age at admission of first operation, sex of the patient, Elixhauser comorbidity score at first operation, annual hospital volume, and the time interval between operations. Variables that were not statistically significant were step-by-step exclude from the model.A *p*-value threshold of 0.05 was considered statistically significant. All data analyses were carried out using R statistical software (Version: R-4.2.0).

## Results

The characteristics of the study population are summarized in Tables 1 and 2. In 7.3% of patients osteoarthritis (OA) of the hip is bilateral and both hips need replacement. 3.8% of all patients who received THA had bilateral procedures within one year. Before matching, 0.3% (*n* = 1,007) of all patients with bilateral THA had their surgery simultenously, 2.4% (*n* = 767) staged in a short interval and 3.2% (*n* = 10,022) staged in an intermediate interval. Mean patient age in the three groups was 62 years (SD 10.7), 64 years (SD 11.4) and 67 years (SD 10.5) respectively (*p* < 0.0001). Mean BMI was lower in the group of simultaneous THA with 26.7 (SD 4.33) compared to 28.1 (SD 5.33) in the group of short and 28.4 (SD 5.39) of intermediate interval (*p* < 0.0001).

**Table 1:** Demographic data of the study population

	Before Matching				After Matching			
	same day	1–90 days	91–365 days	*p*-value	same day	1–90 days	91–365 days	*p*-value
	(*n* = 1007)	(*n* = 767)	(*n* = 10,022)		(*n* = 767)	(*n* = 767)	(*n* = 4602)	
**Age at admission**								
Mean (SD)	61.9 (10.7)	64.4 (11.4)	66.7 (10.5)	< 0.0001	63.0 (10.4)	64.4 (11.4)	64.6 (11.0)	0.001
**Sex of patient**,** n (%)**								
female	564 (56.0%)	446 (58.1%)	6393 (63.8%)	< 0.0001	450 (58.7%)	446 (58.1%)	2689 (58.4%)	0.979
**BMI**								
Mean (SD)	26.7 (4.33)	28.1 (5.33)	28.4 (5.39)	< 0.0001	27.1 (4.59)	28.1 (5.33)	28.2 (5.37)	< 0.001
no information, n (%)	302 (30.0%)	251 (32.7%)	4102 (40.9%)		288 (37.5%)	251 (32.7%)	1506 (32.7%)	
**Elixhauser comorbidity score**								
Mean (SD)	0.652 (3.00)	0.720 (4.15)	0.847 (3.73)	0.206	0.755 (3.28)	0.720 (4.15)	0.746 (3.92)	0.982
**Annual hospital** **volume**,** n (%)**								
[0, 250)	123 (12.2%)	184 (24.0%)	3142 (31.4%)	< 0.0001	89 (11.6%)	184 (24.0%)	1416 (30.8%)	< 0.0001
[250, 500)	201 (20.0%)	220 (28.7%)	3090 (30.8%)		153 (19.9%)	220 (28.7%)	1489 (32.4%)	
[500, …)	676 (67.1%)	349 (45.5%)	3669 (36.6%)		518 (67.5%)	349 (45.5%)	1641 (35.7%)	
no information, n (%)	7 (0.7%)	14 (1.8%)	121 (1.2%)		7 (0.9%)	14 (1.8%)	56 (1.2%)	

*One-way ANOVA for continuous variables (e.g. age at admission), Chi-squared test for categorical variables (e.g. sex of patient)

**Mahalanobis Distance Matching of 1–90 days and same day in a ratio of 1:1, as well as 1–90 days and 91–365 days in a ratio of 1:6 by age at admission at first operation, sex of the patient, BMI at first operation and Elixhauser score (van Walraven variant) at first operation. Perfect balance after matching could not be achieved as groupsize of same day and 1–90 days differed not enough

**Table 2:** Revision and mortality rate

	Before Matching				After Matching			
	same day	1–90 days	91–365 days	*p*-value	same day	1–90 days	91–365 days	*p*-value
	(*n* = 1007; THA = 2014)	(*n* = 767; THA = 1534)	(*n* = 10,022; THA = 20,044)		(*n* = 767; THA = 1534)	(*n* = 767; THA = 1534)	(*n* = 4602; THA = 9204)	
**Revision (any THA)**								
	23 (1.1%)	24 (1.6%)	388 (1.9%)	0.029	22 (1.4%)	24 (1.6%)	193 (2.1%)	0.112
**Revision of 1st THA**,** n (%)**								
all		5 (0.7%)	138 (1.4%)	0.221	12 (1.6%)	5 (0.7%)	68 (1.5%)	0.175
septic		2 (0.3%)	50 (0.5%)	0.462	3 (0.4%)	2 (0.3%)	27 (0.6%)	0.442
**Time to revision of 1st THA**,** n (%)**								
on ward (0–10 days)	3 (0.3%)	1 (0.1%)	29 (0.3%)	0.264	3 (0.4%)	1 (0.1%)	17 (0.4%)	0.189
11 days to 3 months	7 (0.7%)	1 (0.1%)	64 (0.6%)		7 (0.9%)	1 (0.1%)	34 (0.7%)	
3–12 months	2 (0.2%)	1 (0.1%)	11 (0.1%)		2 (0.3%)	1 (0.1%)	5 (0.1%)	
No revision	995 (98.8%)	761 (99.2%)	9882 (98.6%)		755 (98.4%)	761 (99.2%)	4534 (98.5%)	
**Revision of 2nd THA**,** n (%)**								
all	11 (1.1%)	19 (2.5%)	250 (2.5%)	0.020	10 (1.3%)	19 (2.5%)	125 (2.7%)	0.068
septic	4 (0.4%)	6 (0.8%)	87 (0.9%)	0.286	3 (0.4%)	6 (0.8%)	40 (0.9%)	0.387
**Time to revision of 2nd THA**,** n (%)**								
on ward (0–10 days)	1 (0.1%)	2 (0.3%)	38 (0.4%)	0.185	1 (0.1%)	2 (0.3%)	16 (0.3%)	0.173
10 days to 3 months	0 (0%)	0 (0%)	0 (0%)		0 (0%)	0 (0%)	0 (0%)	
3–12 months	2 (0.2%)	5 (0.7%)	30 (0.3%)		1 (0.1%)	5 (0.7%)	19 (0.4%)	
no information, n (%)	997 (99.0%)	756 (98.6%)	9912 (98.9%)		758 (98.8%)	756 (98.6%)	4547 (98.8%)	
**Death of patient after 2nd THA**,** n (%)**								
	19 (1.9%)	18 (2.3%)	301 (3.0%)	0.087	17 (2.2%)	18 (2.3%)	121 (2.6%)	0.745
**Time to death after2nd THA**,** n (%)**								
alive	988 (98.1%)	749 (97.7%)	9721 (97.0%)	0.222	750 (97.8%)	749 (97.7%)	4481 (97.4%)	0.637
within 1 year	4 (0.4%)	3 (0.4%)	71 (0.6%)		2 (0.3%)	3 (0.4%)	31 (0.7%)	
no information, n (%)	1 (0.1%)	0 (0%)	4 (0.0%)		1 (0.1%)	0 (0%)	1 (0.0%)	

*One-way ANOVA for continuous variables (e.g. age at admission), Chi-squared test for categorical variables (e.g. sex of patient)

**Mahalanobis Distance Matching of 1–90 days and same day in a ratio of 1:1, as well as 1–90 days and 91–365 days in a ratio of 1:6 by age at admission at first operation, sex of the patient, BMI at first operation and Elixhauser score (van Walraven variant) at first operation. Perfect balance after matching could not be achieved as groupsize of same day and 1–90 days differed not enough

67.1% of the patients underwent simultaneous THA in a high-volume center with more than 500 THA per year, while 20.0% had their surgery in a center with 250 to 500 surgeries and 12.2% in a low-volume center with just up to 250 surgeries.

After matching, the study groups consisted of 767 patients who had simultaneous THA, 767 patients in short and 4,602 patients in intermediate interval.

No significant differences were seen between sex and the number of comorbidities (Elixhauser score) between the three groups. Patients with just one unilateral THA who are included in the EPRD were older (mean 69.4 years), more female patients (62%) and with severe comorbidities (Elixhauser score 1.43; SD 4.33).

**Fig. 2:**
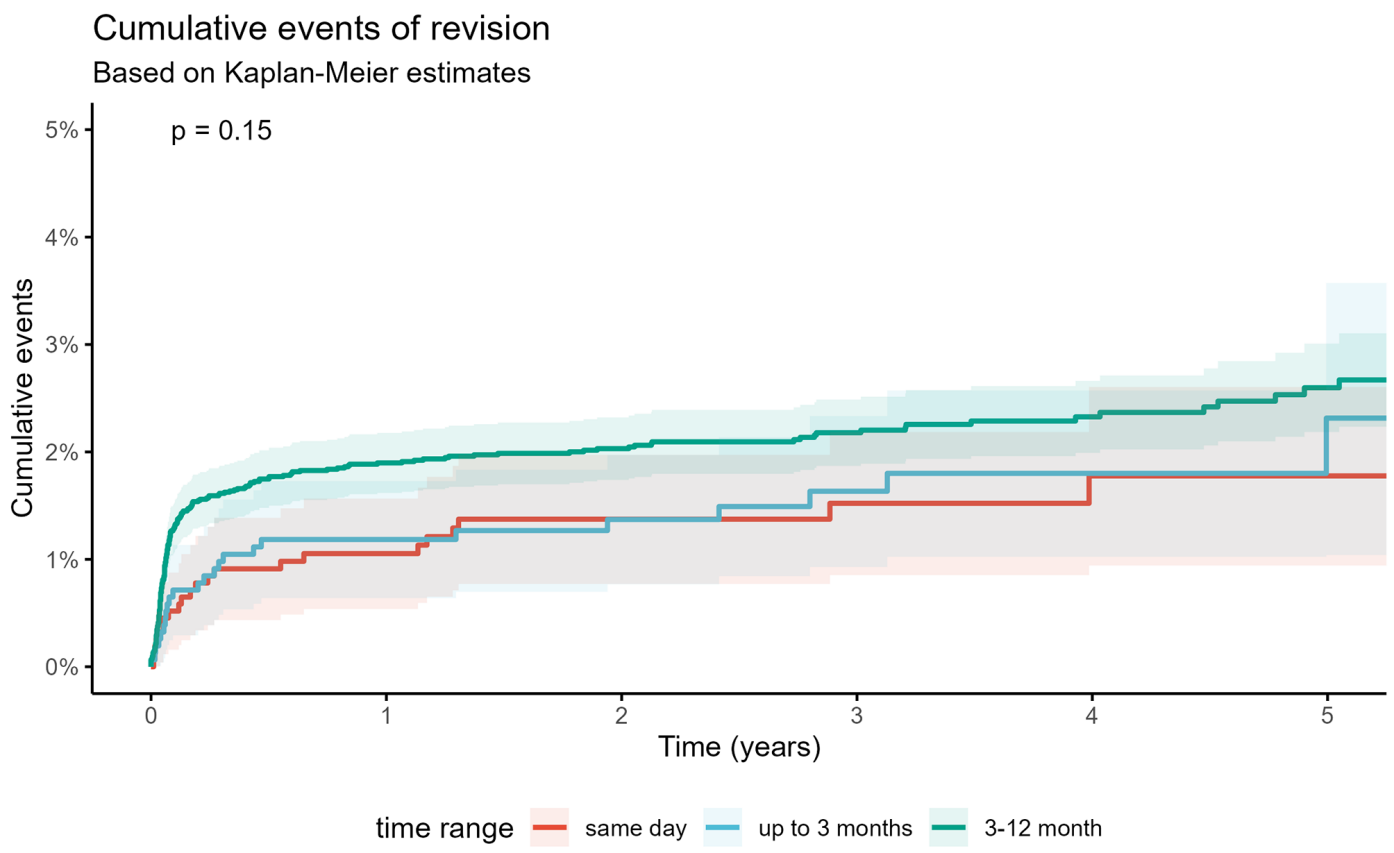
Revision after any THA (Kaplan-Meier)

The analysis demonstrated a low cumulative revision rate in every group, see Fig. 2.

Patients with simultaneous THA had a lower cumulative revision rate of 1.8% (95% CI 0.9–2.6) within five years in comparison to two-staged THA in short interval with 2.3% (95% CI 1.0–3.6; *p* = 0.760) and intermediate interval with 2.5% (95% CI 2.1–2.96; *p* = 0.310) see Table 3. With missing data up to 33% there were no significant differences between regarding reasons of revision. We found 2.0%, 3.02% and 2.9% aseptic and 0.8%, 1.4% and 1.5% septic revisions in the three different groups. Most common reason for revision in same day surgeries were periprosthetic fractures (33% for 1st and 0% for 2nd THA), in the short interval infection (20% and 26%) and in the intermediate interval infection as well (23% and 23%), see Table 4.

**Table 3:** Cumulative events for revision (95% confidence interval)

	90 days	1 year	2 years	3 years	4 years	5 years
**same day (%)**	0.9 (0.4–1.3)	1.1 (0.5–1.6)	1.4 (0.8-2.0)	1.5 (0.9–2.2)	1.8 (0.9–2.6)	1.8 (0.9–2.6)
**1–90 days (%)**	0.9 (0.4–1.3)	1.2 (0.6–1.7)	1.4 (0.8-2.0)	1.6 (0.9–2.4)	1.8 (1.0-2.6)	2.3 (1.0-3.6)
**91–365 days (%)**	1.5 (1.3–1.8)	1.8 (1.5–2.1)	1.9 (1.6–2.2)	2.1 (1.8–2.4)	2.2 (1.9–2.6)	2.5 (2.1–2.9)

**Table 4:** Reason for revision

	Before Matching				After Matching			
	same day	1–90 days	91–365 days	*p*-value	same day	1–90 days	91–365 days	*p*-value
**aseptic reason**								
1st THA	9 (0.9%)	3 (0.4%)	88 (0.9%)	0.4	9 (1.2%)	3 (0.4%)	42 (0.9%)	0.2
2nd THA	7 (0.7%)	13 (1.7%)	163 (1.6%)	0.071	7 (0.9%)	13 (1.7%)	92 (2.0%)	0.11
**septic reason**								
1st THA	3 (0.3%)	2 (0.3%)	50 (0.5%)	0.6	3 (0.4%)	2 (0.3%)	27 (0.6%)	0.6
2nd THA	4 (0.4%)	6 (0.8%)	87 (0.9%)	0.3	3 (0.4%)	6 (0.8%)	43 (0.9%)	0.3
**reason for revision of 1st THA**								
Condition after removal	0 (0%)	0 (0%)	2 (1.4%)	0.3	0 (0%)	0 (0%)	1 (1.4%)	0.5
Dislocation	0 (0%)	2 (40%)	12 (8.7%)		0 (0%)	2 (40%)	6 (8.7%)	
Infection	2 (17%)	1 (20%)	34 (25%)		2 (17%)	1 (20%)	16 (23%)	
Loosening (Cup)	0 (0%)	0 (0%)	11 (8.0%)		0 (0%)	0 (0%)	5 (7.2%)	
Loosening (Stem)	1 (8.3%)	0 (0%)	7 (5.1%)		1 (8.3%)	0 (0%)	4 (5.8%)	
Malalignment	0 (0%)	0 (0%)	2 (1.4%)		0 (0%)	0 (0%)	1 (1.4%)	
Other reasons	2 (17%)	0 (0%)	11 (8.0%)		2 (17%)	0 (0%)	7 (10%)	
Periprosthetic fracture	4 (33%)	1 (20%)	12 (8.7%)		4 (33%)	1 (20%)	6 (8.7%)	
Missing	3 (25%)	1 (20%)	47 (34%)		3 (25%)	1 (20%)	23 (33%)	
**reason for revision of 1st THA**								
Component failure	0 (0%)	0 (0%)	1 (0.4%)	0.7				0.7
Condition after removal	0 (0%)	0 (0%)	5 (2.0%)		0 (0%)	0 (0%)	3 (2.2%)	
Dislocation	1 (9.1%)	1 (5.3%)	25 (10%)		1 (10%)	1 (5.3%)	16 (12%)	
Infection	3 (27%)	5 (26%)	64 (26%)		2 (20%)	5 (26%)	31 (23%)	
Loosening (Cup and stem)	0 (0%)	0 (0%)	1 (0.4%)					
Loosening (Cup)	2 (18%)	1 (5.3%)	18 (7.2%)		2 (20%)	1 (5.3%)	13 (9.6%)	
Loosening (Stem)	3 (27%)	1 (5.3%)	15 (6.0%)		3 (30%)	1 (5.3%)	9 (6.7%)	
Malalignment	0 (0%)	1 (5.3%)	4 (1.6%)		0 (0%)	1 (5.3%)	2 (1.5%)	
Osteolysis with fixed component (Stem)	0 (0%)	0 (0%)	1 (0.4%)					
Other reasons	1 (9.1%)	2 (11%)	22 (8.8%)		1 (10%)	2 (11%)	13 (9.6%)	
Periprosthetic fracture	0 (0%)	1 (5.3%)	30 (12%)		0 (0%)	1 (5.3%)	13 (9.6%)	
Wear	0 (0%)	0 (0%)	5 (2.0%)		0 (0%)	0 (0%)	5 (3.7%)	
Missing	1 (9.1%)	7 (37%)	59 (24%)		1 (10%)	7 (37%)	30 (22%)	

*One-way ANOVA for continuous variables (e.g. age at admission), Chi-squared test for categorical variables (e.g. sex of patient)

**Mahalanobis Distance Matching of 1–90 days and same day in a ratio of 1:1, as well as 1–90 days and 91–365 days in a ratio of 1:6 by age at admission at first operation, sex of the patient, BMI at first operation and Elixhauser score (van Walraven variant) at first operation. Perfect balance after matching could not be achieved as groupsize of same day and 1–90 days differed not enough

After adjusting for age, sex, comorbidities in any time interval, and annual hospital volume, patients with bilateral THA in a high-volume center (≥ 500 THA per year) had a significant lower risk for revision (HR 0.687; 95% CI 0.501–0.942) compared to surgeries in a low-volume center (< 250 THA per year). For bilateral surgeries in a medium-volume center (250–500 THA per year) there was no difference (HR 0.974; 95% CI 0.713–1.331). The variables age at admission, sex of the patient, Elixhauser comorbidity score and time interval between both operations were not statistically significant and consequently excluded from the model.

**Fig. 3:**
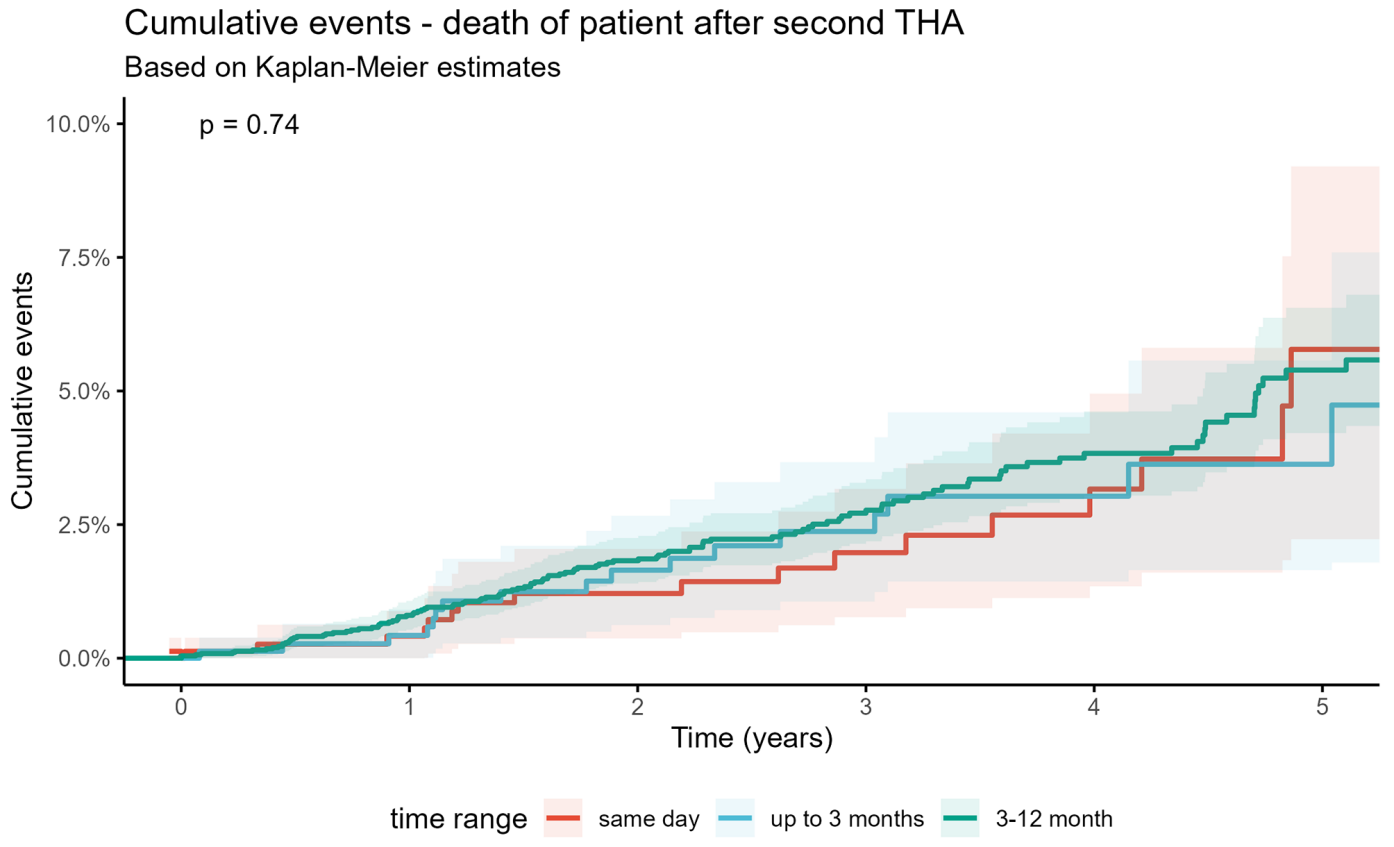
Death of patient after second THA (Kaplan-Meier)

There was no significant difference between the cumulative mortality in every group, see Fig. 3. After five years the cumulative mortality rate was 5.8% (95% CI 2.2–9.3) for patients who had simultaneous THA, 3.7% (95% CI 1.7–5.6) for patients with two-staged THA in the short interval group and 5.3% (95% CI 4.1–6.5) in the intermediate interval group, respectively (Table 5). Higher age (HR 1.060; 95% CI 1.042–1.078) and severe comorbidities, Elixhauser Score (HR 1.046; 95% CI 1.014–1.079) were associated with higher mortality rates after simultaneous THA. Female sex was associated with lower mortality (HR 0.682; 95% CI 0.494–0.943) in this group. As annual hospital volume and THA interval did not have a statistically significant influence, both variables were consequently excluded from the model.

**Table 5:** Cumulative events for death of patient after second THA (95% confidence interval)

	90 days	1 year	2 years	3 years	4 years	5 years
**same day (%)**	0.1 (0.0-0.4)	0.4 (0.0-0.9)	1.2 (0.4–2.1)	2.0 (0.8–3.2)	3.2 (1.4-5.0)	5.8 (2.2–9.3)
**1–90 days (%)**	0.1 (0.0-0.4)	0.4 (0.0-0.9)	1.7 (0.6–2.7)	2.4 (1.1–3.7)	3.1 (1.4–4.6)	3.7 (1.7–5.6)
**91–365 days (%)**	0.1 (0.0-0.2)	0.8 (0.5-1.0)	1.8 (1.4–2.2)	2.7 (2.2–3.3)	3.7 (3.0-4.5)	5.3 (4.1–6.5)

## Discussion

7.3% of patients with primary hip replacement, who are documented in the German Arthroplasty Registry (EPRD), have had both hip joints replaced, 3.8% in the same year and 0.3% simultaneously. This is less than reported in other registries with 1.9% in New Zealand and 0.8% in Australia [20, 21]. Due to the ongoing discussion about the safety of simultaneous bilateral surgery we evaluated the risks of mortality and revision rates in these cohorts and compared them with patients, who had staged surgery in longer intervals.

In several studies benefits as well as risks of simultaneous THA compared to different two-staged surgery strategies have been compared. Although there are some investigations, where more complications have been reported after simultaneous THA [4, 14–16], others did not see a difference between bilateral and unilateral staged surgery [12, 13, 22].

Many studies are focusing on perioperative complications and procedure-related characteristics (i.e. length of stay) in relatively small patient cohorts from single institutions. In addition, most of them are comparing simultaneous surgery with staged procedures independently from the interval between index and contralateral THA. In contrast, we have analyzed mortality as well as revision rates in a large national data set and compared patients with surgery in different time intervals. We found a low cumulative revision rate in every group. Patients with simultaneous THA had a lower cumulative revision rate compared to two-staged THA.

In a high sample study from the Australian Registry about bilateral THA with different intervals, Calabro et al. reported about similar long-term revision rates. Two-staged THA between three and six months had the lowest risk for revision. The reasons for revision were similar in all groups with fracture followed by loosening and infection. Simultaneous bilateral THA had a significantly higher rate for fracture compared to staged bilateral THA [20]. Wyatt et al. reported data from the New Zealand Joint Registry and found the highest rate of revision for two-staged THA in the short interval within 90 days, most commonly for cup loosening. Revision causes infection and dislocation were similar in all groups [21].

Garland et al. reported from the Swedish Hip Arthroplasty Register and indicated a slightly higher, but unadjusted risk for revision for two-staged THA, but without information about causes. After adjusting for sex, age, diagnosis and type of prosthesis fixation, this difference in the risk estimates disappeared [23].

Ramezani et al. concluded from a systematic review and meta-analysis with an increased risk for periprosthetic fracture in simultaneous bilateral THA and comparable risk for periprosthetic joint infection and dislocation [16]. As in prior studies we could confirm that patients undergoing simultaneous THA were often younger [4, 15, 16, 22, 24] and in addition, Calabro et al. discussed, that in simultaneous THA more commonly cementless stems were used. This may be a reason for the higher revision rate for fracture in simultaneous THA [20].

Regarding the reported complications, there is a wide variability in the results, due to different study designs and heterogeneity of control groups. However, several authors mention a volume-outcome-relationship, which may not only affect the results after unilateral arthroplasty procedures but also can have an impact on the outcome after bilateral surgery. After five years the lowest cumulative revision rate in our patients was seen in the group who underwent both THA simultaneously and who underwent their bilateral THA in a high-volume center (≥ 500 THA per year). Similar results were seen in unilateral THA with higher revision rates (4.3% at 5year-FU) in hospitals with < 250 THA per year compared to lower revision rates (3.3% at 5y-FU) in hospitals with ≥ 500 THA per year in Germany [25]. Tsiridis et al., who performed an early meta-analysis of published studies up to 2006, already recommended, that simultaneous THA should be ideally undertaken in tertiary referral hospitals which are more experienced in major hip surgery [26]. Partridge et al. excluded unilateral THA and concluded from their data, that fewer complications were seen in centers that perform simultaneous procedures more routinely, possibly because all healthcare professionals will be familiar with the whole treatment. Additionally, their patients in the high-volume group were significantly younger and less comorbid [15]. Regarding high-volume centers, Partridge et al. had chosen a threshold of five simultaneous THA per year, which resulted in a significant difference of overall complication rates [15]. Najfeld et al. reported in a study from another high-volume-center, that simultaneous THA or total knee arthroplasty (TKA) is not associated with significant differences in complications, readmission rate up to 30 days or higher mortality rate [24].

One further study showed a significantly increased mortality risk for simultaneous THA, if not treated in a high volume center, in comparison to a staged group and the national average for unilateral THA in the UK [15]. Garland et al. evaluated the Swedish Hip Arthroplasty Register and described an elevated early postoperative mortality rate within 90 days associated with factors like advanced age, RA, high ASA class and male sex [23]. In this study female patients had lower mortality rates, while sex showed no association with revision. Mortality was lower in our cohort for bilateral THA than for unilateral THA in the same time frame (9.6%) or than in reported studies, which underlines that bilateral procedures are reserved for patients in younger age and healthier conditions [25, 27].

### Limitations

Our study design has some weaknesses. Main limitation is, that this is a registry related study without nationwide indications for bilateral or staged bilateral THA. At the same time, we have only analyzed mortality and revision rates, without addressing some confounders as type of implants, detailed reasons for revision e.g., which do not give an overall picture of peri- and post-operative complications in detail without consecutive surgery or death. Nevertheless, the cohort size is very large and non-surgical complications are not reported in most national registries.

As the EPRD is a voluntary registry, not all THA performed in Germany are included. Patients with bilateral OA, who died after their first THA, were lost as well. However, data can be considered representative as the registry covers about 70% of all arthroplasties performed in Germany. In addition, most hospitals which do not enter data into the EPRD, are small hospitals which very unlikely perform bilateral THA. The strength of the current study is the first midterm analysis up to five years with a large sample size, which is higher than all published meta-analysis and registry studies. Furthermore, the nearly complete follow-up allows for an estimation of valid real-world data.

## Conclusions

Simultaneous bilateral THA seems to be a safe procedure for younger patients with limited comorbidities who have bilateral end-stage hip OA, especially if performed in high-volume centers. These factors should be considered in shared-decision making about simultaneous or staged bilateral THA.

## Data Availability

All data generated or analyzed during this study are included in this published article.

## Abbreviations

CI: Confidence interval
ICD-10: International classification of disease
OA: Osteoarthritis
HR: Hazard Ratio
THA: Total Hip Arthroplasty
TKA: Total Knee Arthroplasty
TJA: Total Joint Arthroplasty

## References

[1] Learmonth ID, Young C, Rorabeck C. The operation of the century: total hip replacement. Lancet. 2007;370(9597):1508–19. 17964352 10.1016/S0140-6736(07)60457-7

[2] Evans JT, Evans JP, Walker RW, Blom AW, Whitehouse MR, Sayers A. How long does a hip replacement last? A systematic review and meta-analysis of case series and national registry reports with more than 15 years of follow-up. Lancet. 2019;393(10172):647–54. 30782340 10.1016/S0140-6736(18)31665-9 PMC6376618

[3] Callhoff J, Albrecht K, Redeker I, Lange T, Goronzy J, Günther KP, Zink A, Schmitt J, Saam J, Postler A. Disease burden of patients with osteoarthritis: results of a cross-sectional survey linked to claims data. Arthritis Care Res. 2020;72(2):193–200. 10.1002/acr.24058 31479193

[4] Almaguer A, Cichos K, McGwin G Jr, Pearson J, Wilson B, Ghanem E. Combined total hip and knee arthroplasty during the same hospital admission: is it safe? Bone Joint J. 2019;101(5):573–81. 31038999 10.1302/0301-620X.101B5.BJJ-2018-1438

[5] Jaffe W, Charnley J. Bilateral Charnley low-friction arthroplasty as a single operative procedure. A report of fifty cases. Bull Hosp Joint Dis. 1971;32(2):198–214. 5128234

[6] Ritter MA, Stringer EA. Bilateral total hip arthroplasty: a single procedure. Clin Orthop Relat Res 1980(149):185–90. 7408301

[7] Cammisa FP Jr, O’Brien SJ, Salvati EA, Sculco TP, Wilson PD Jr, Ranawat CS, Pellicci PM, Inglis AE. One-stage bilateral total hip arthroplasty: a prospective study of perioperative morbidity. Orthop Clin North Am. 1988;19(3):657–68. 3380539 10.1016/S0030-5898(20)32272-0

[8] Berend ME, Ritter MA, Harty LD, Davis KE, Keating EM, Meding JB, Thong AE. Simultaneous bilateral versus unilateral total hip arthroplasty: an outcomes analysis. J Arthroplast. 2005;20(4):421–6. 10.1016/j.arth.2004.09.062 16124956

[9] Eggli S, Huckell C, Ganz R. Bilateral total hip arthroplasty: one stage versus two stage procedure. Clin Orthop Relat Res (1976–2007). 1996;328:108–18. 10.1097/00003086-199607000-00019 8653943

[10] Alfaro-Adrian J, Bayona F, Rech J, Murray D. One-or two-stage bilateral total hip replacement. J Arthroplast. 1999;14(4):439–45. 10.1016/S0883-5403(99)90099-2 10428224

[11] Bhan S, Pankaj A, Malhotra R. One-or two-stage bilateral total hip arthroplasty: a prospective, randomised, controlled study in an Asian population. J Bone Joint Surg Br Volume. 2006;88(3):298–303. 10.1302/0301-620X.88B3.17048 16498000

[12] Parvizi J, Tarity TD, Sheikh E, Sharkey PF, Hozack WJ, Rothman RH. Bilateral total hip arthroplasty: one-stage versus two-stage procedures. Clin Orthop Relat Research^®^. 2006;453:137–41. 10.1097/01.blo.0000246529.14135.2b 17312590

[13] Reuben JD, Meyers SJ, Cox DD, Elliott M, Watson M, Shim SD. Cost comparison between bilateral simultaneous, staged, and unilateral total joint arthroplasty. J Arthroplast. 1998;13(2):172–9. 10.1016/S0883-5403(98)90095-X 9526210

[14] Flick TR, Ofa SA, Patel AH, Ross BJ, Sanchez FL, Sherman WF. Complication rates of bilateral total hip versus unilateral total hip arthroplasty are similar. J Orthop. 2020;22:571–8. 33299269 10.1016/j.jor.2020.11.010 PMC7688989

[15] Partridge TC, Charity JA, Sandiford NA, Baker PN, Reed MR, Jameson SS. Simultaneous or staged bilateral total hip arthroplasty? An analysis of complications in 14,460 patients using national data. J Arthroplast. 2020;35(1):166–71. 10.1016/j.arth.2019.08.022 31521445

[16] Ramezani A, Ghaseminejad Raeini A, Sharafi A, Sheikhvatan M, Mortazavi SMJ, Shafiei SH. Simultaneous versus staged bilateral total hip arthroplasty: a systematic review and meta-analysis. J Orthop Surg Res. 2022;17(1):1–25. 35964047 10.1186/s13018-022-03281-4 PMC9375332

[17] Grimberg A, Jansson V, Lützner J, Melsheimer O, Morlock M. Steinbrück A: EPRD Jahresbericht 2021; 2021.

[18] Jansson V, Grimberg A, Melsheimer O, Perka C, Steinbrück A. Orthopaedic registries: the German experience. EFORT Open Reviews. 2019;4(6):401–8. 31210976 10.1302/2058-5241.4.180064 PMC6549118

[19] van Walraven C, Austin PC, Jennings A, Quan H, Forster AJ. A modification of the Elixhauser comorbidity measures into a point system for hospital death using administrative data. Med Care 2009:626–33. 10.1097/MLR.0b013e31819432e5 19433995

[20] Calabro L, Yong M, Whitehouse SL, Hatton A, de Steiger R, Crawford RW. Mortality and implant survival with simultaneous and staged bilateral total hip arthroplasty: experience from the Australian orthopedic association national joint replacement registry. J Arthroplast. 2020;35(9):2518–24. 10.1016/j.arth.2020.04.027 32402580

[21] Wyatt MC, Hozack JW, Frampton C, Rothwell A, Hooper GJ. Is single-anaesthetic bilateral primary total hip replacement still safe? A 16-year cohort study from the New Zealand Joint Registry. ANZ J Surg. 2018;88(12):1289–93. 30347492 10.1111/ans.14864

[22] Pfeil J, Höhle P, Rehbein P. Bilateral endoprosthetic total hip or knee arthroplasty. Deutsches Ärzteblatt International. 2011;108(27):463. 21814521 10.3238/arztebl.2011.0463 PMC3147284

[23] Garland A, Rolfson O, Garellick G, Kärrholm J, Hailer NP. Early postoperative mortality after simultaneous or staged bilateral primary total hip arthroplasty: an observational register study from the Swedish hip Arthroplasty Register. BMC Musculoskelet Disord. 2015;16(1):1–9. 25887667 10.1186/s12891-015-0535-0 PMC4393879

[24] Najfeld M, Kalteis T, Spiegler C, Ley C, Hube R. The safety of bilateral simultaneous hip and knee arthroplasty versus staged arthroplasty in a high-volume Center comparing blood Loss, peri-and postoperative complications, and early functional outcome. J Clin Med. 2021;10(19):4507. 34640522 10.3390/jcm10194507 PMC8509744

[25] Grimberg A, Jansson V, Lützner J, Melsheimer O, Morlock M, Steinbrück A. EPRD-Jahresbericht 2020. EPRD Deutsche Endoprothesenregister Berlin; 2020.

[26] Tsiridis E, Pavlou G, Charity J, Tsiridis E, Gie G, West R. The safety and efficacy of bilateral simultaneous total hip replacement: an analysis of 2063 cases. J Bone Joint Surg Br Volume. 2008;90(8):1005–12. 10.1302/0301-620X.90B8.20552 18669954

[27] Turan O, Pan X, Kunze KN, Rullan PJ, Emara AK, Molloy RM, Piuzzi NS. 30-day to 10-year mortality rates following total hip arthroplasty: a meta-analysis of the last decade (2011–2021). Hip Int. 2024;34(1):4–14. 36705090 10.1177/11207000231151235

